# Clinical and molecular features of Epstein‐Barr virus‐positive diffuse large B‐cell lymphoma: Results in a multi‐center trial

**DOI:** 10.1002/ctm2.539

**Published:** 2021-09-16

**Authors:** Chen Xing Zhao, Jing Jing Wen, Di Fu, Peng Peng Xu, Shu Cheng, Li Wang, Chao Fu Wang, Xiao Chun Fei, Xin Wang, Jian Feng Zhou, Li Ping Su, Zhuo Wen Chen, Jie Ping Chen, Mei Yun Fang, Ting Liu, Yong Ping Song, Kang Yu, Yan Li, Jian Gu, Ming Hou, Wei Li Zhao, Jian da Hu

**Affiliations:** ^1^ Fujian Provincial Key Laboratory of Hematology Fujian Institute of Hematology Fujian Medical University Union Hospital Fuzhou China; ^2^ State Key Laboratory of Medical Genomics National Research Center for Translational Medicine at Shanghai Shanghai Institute of Hematology Ruijin Hospital Affiliated to Shanghai Jiao Tong University School of Medicine Shanghai China; ^3^ Department of Hematology Shandong Provincial Hospital Affiliated to Shandong University Jinan China; ^4^ Department of Hematology Tongji Medical College Huazhong University of Science and Technology Tongji Hospital Wuhan China; ^5^ Department of Hematology Shanxi Provincial Cancer Hospital Taiyuan China; ^6^ Department of Hematology the First People's Hospital of Foshan Foshan China; ^7^ Department of Hematology Southwestern Hospital Third Military Medical University Chongqing China; ^8^ Department of Hematology First Hospital of Dalian Medical University Dalian China; ^9^ Department of Hematology Hematology Research Laboratory West China Hospital Sichuan University Chengdu China; ^10^ Department of Hematology Henan Cancer Hospital the Affiliated Cancer Hospital of Zhengzhou University Zhengzhou China; ^11^ Department of Hematology the First Affiliated Hospital of Wenzhou Medical University Wenzhou China; ^12^ Department of Hematology the First Hospital of China Medical University Shenyang China; ^13^ Institute of Hematology Subei People's Hospital of Jiangsu Province Clinical Medical College of Yangzhou University Yangzhou China; ^14^ Department of Hematology Qilu Hospital Shandong University Jinan China; ^15^ Pôle de Recherches Sino‐Français en Science du Vivant et Génomique Laboratory of Molecular Pathology Shanghai China

Dear Editor,

Epstein‐Barr virus (EBV) is an important human oncogenic virus and closely related to the pathogenesis of diffuse large B‐cell lymphoma.^1^ This is, to our knowledge, the first study to systematically evaluate the clinical impact of EBV infection on DLBCL, based on a prospective clinical trial (NHL‐001, NCT01852435). Meanwhile, genomic and transcriptomic features of EBV+DLBCL were further investigated by whole exome/genome sequencing (WES/WGS), targeted‐, and RNA‐sequencing.

EBV‐encoded RNA (EBER) in situ hybridization is the gold standard of EBV‐infection status in tumors.^2^ Here 46 of 429 patients were positive for tumor EBER (10.7%) and EBER‐positive patients had poor performance status (ECOG≥2, 35.9% vs 11.8%, *P* = 0.0338) and low rate of complete remission (CR, 45.7% vs 88.8%, *P* < 0.0001) (Table 1). In univariate analysis (Figure 1A), adverse prognostic factors for both progression‐free survival (PFS) and overall survival (OS) included age, ECOG≥2, advanced stage, extranodal site≥2, elevated serum LDH, IPI > 2, and EBER positivity. In multivariate analysis, EBER positivity independently indicated adverse outcome (*P* = 0.0328 for PFS and *P* = 0.0182 for OS, Table S1). The 2‐year PFS and OS were 47.8% and 69.6% for EBER‐positive patients, and 86.4% and 92.2% for EBER‐negative patients (*P* both < 0.0001, Figure 1B). This was observed not only in young patients (*P* both < 0.0001, Figure 1C), but also in elderly patients (*P* both < 0.0001, Figure 1D). The adverse prognostic effect of EBV infection was in consistent with cohorts from Japan,^3^ and Korea,^4^ but not in western countries.^5^ probably due to relatively low incidence of EBV infection in western population. Nevertheless, EBER‐positive DLBCL should be referred to an individual clinical entity in both younger and elderly patients.

**TABLE 1:** Clinical characteristics of DLBCL patients according to tumor EBER

	EBER		
Characteristics	Pos	Neg	*P*
	*N *= 46	*N *= 383	
**Age**			0.3224
>60	18 (39.13)	122 (31.85)	
≤60	28 (60.87)	261 (68.15)	
**Sex**			0.0596
Male	31 (67.39)	198 (51.70)	
Female	15 (32.61)	185 (48.30)	
**ECOG**			0.0338
0–1	35 (76.09)	338 (88.25)	
2–5	11 (23.91)	45 (11.75)	
**Ann‐Arbor stage**			0.3531
I/II	22 (47.83)	211 (55.09)	
III/IV	24 (52.17)	172 (44.91)	
**Extranodal site**			0.5742
0–1	34 (73.91)	299 (78.07)	
≥2	12 (26.09)	84 (21.93)	
**LDH**			0.1472
Elevated	21 (45.65)	133 (34.73)	
Normal	25 (54.35)	250 (65.27)	
**IPI**			0.2733
0–2	32 (69.57)	295 (77.02)	
3–5	14 (30.43)	88 (22.98)	
**Hans** *			1.0000
GCB	19 (44.19)	156 (44.07)	
Non‐GCB	24 (55.81)	198 (55.93)	
**CR**			0.0000
Yes	21 (45.65)	340 (88.77)	
No	25 (54.35)	43 (11.23)	

Values are reported as *N* (%) of patients unless indicated otherwise.

* The calculation was based on 397 patients with available data. CR, complete remission; ECOG, Eastern Cooperative Oncology Group IPI, International Prognostic Index; LDH, lactic dehydrogenase; GCB, Germinal center B‐cell; Neg, negative; Pos, positive.

**FIGURE 1:**
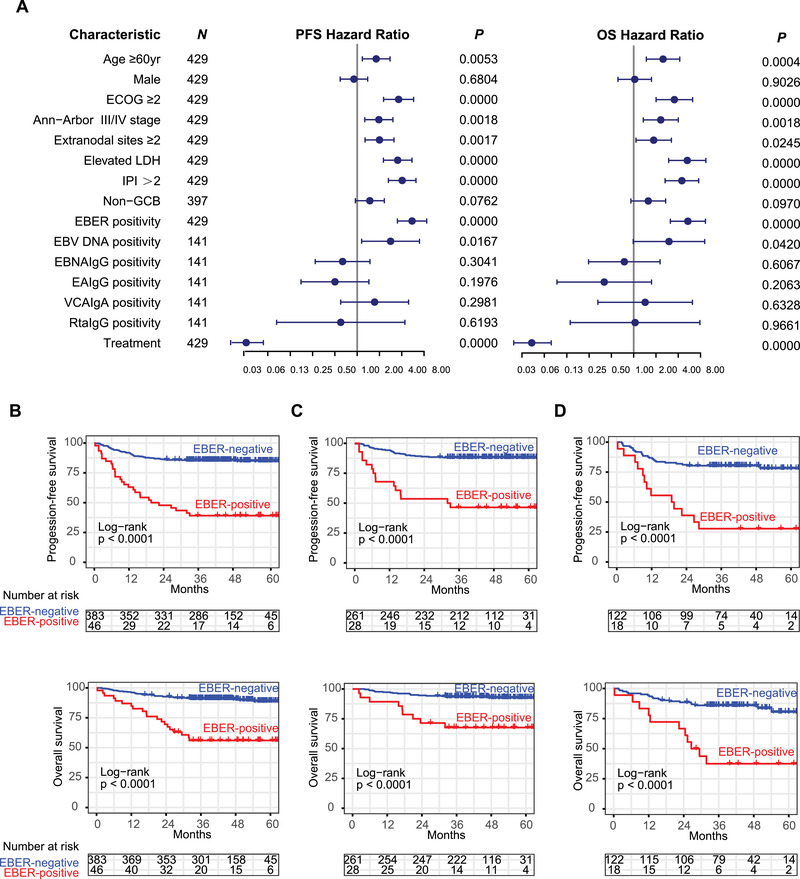
Survival analysis in DLBCL patients according to tumor EBER. (A) Forest plot of PFS and OS in DLBCL patients according to different clinical characteristics. (B) Survival curves of PFS and OS for all patients, (C) for young patients and (D) for elderly patients according to tumor EBER. PFS, progression‐free survival; OS, overall survival; HR, Hazard ratio; CI, confidence interval; GCB, germinal center B‐cell; ECOG, Eastern Cooperative Oncology Group; IPI, International Prognostic Index; LDH, lactic dehydrogenase

As for EBV‐associated serum markers, EBV‐DNA is usually negligible in cell‐free body fluid like serum, but becomes detectable in EBV‐related disease.^2^ Among 141 patients available for serum samples, serum EBV DNA were positive in 14 cases, correlated with tumor EBER (*P* = 0.0036, concordance rate 82.3%, Table S2) and shared similar impact on ECOG≥2 (35.7% vs 11.81% *P* = 0.0298) and response to immunochemotherapy (CR, 64.3% vs 89.8%, *P* = 0.0186, Table S3). In univariate analysis, serum EBV DNA predicted unfavorable 2‐year PFS (66.7% vs 84.2%, *P* = 0.0167) and 2‐year OS (72.2% vs 92.1%, *P* = 0.0420). As serum EBV DNA is mostly derived from tumor cells,^6^ our findings emphasized the advantage of serum EBV DNA as noninvasive biomarker for predicting tumor EBER status and prognostic factor of DLBCL. EBV serological tests refer to different humoral antibody patterns against viral proteins, including Epstein‐Barr nuclear antigen (EBNA), early antigen (EA), viral capsid antigen (VCA) and Rta protein (Rta). Among these 141 patients, 116 cases were positive for EBNA IgG (116/141, 82.3%), 33 cases positive for EA IgG (33/141, 23.4%), 12 cases for VCA IgA (12/141, 8.5%), and 8 cases for Rta IgG (8/141, 5.7%). Although EBV serological markers were not correlated with tumor EBER (Table S2) and prognosis (Figure 1A), VCA IgA‐positive patients tend to have elevated serum LDH (67.4% vs 33.3%, *P* = 0.0264) and higher IPI (58.8% vs 22.2%, *P* = 0.0022, Table S3), providing additional clinical information in EBV‐related DLBCL.

Genomic alterations play an important role on pathogenesis of EBV‐associated malignancies.^7^ Here, by integrating WES/WGS, targeted‐ and RNA‐sequencing, we described a comprehensive view of genomic alterations based on a large cohort of DLBCL. Gene mutations were first screened in 180 patients with WGS/WES/targeted sequencing data, including 40 cases of EBER‐positive DLBCL and 140 cases of EBER‐negative DLBCL. No significant difference was observed in EBER‐positive DLBCL according to cell of origin or genotypes (Table S4). Seven highly mutated genes were identified (Figure 2A), including *DDX3X* (22.5% vs 9.3%), *TET2* (22.5% vs 7.1%), *MYC* (20.0% vs 5.0%), *STAT3* (17.5% vs 3.6%), *TNFAIP3* (17.5% vs 3.6%), *TNFRSF14* (15.0% vs 4.3%), and *LYN* (10.0% vs 1.4%) (Figures S1 and 2B). Previously revealed by targeted sequencing, epigenetic regulator *TET2* and transcription factor *MYC* were frequently mutated in EBV + DLBCL. We not only confirmed the association of *TET2* and *MYC* mutations with EBV infection in DLBCL, but also revealed significant enrichment of *TET2*‐ and *MYC*‐targeted genes expression in EBER‐positive DLBCL (Figure 2C). As for major signaling pathways (Figure 2B), EBER‐positive DLBCL showed aberrant activation of Wnt, NF‐κB, and JAK‐STAT pathway and inhibition of BCR signaling pathway, as compared to EBER‐negative DLBCL (Figure 2C). It is possible that the mutation changes in these pathways promote tumor cell proliferation and progression in EBER‐positive DLBCL in a BCR‐signaling‐independent manner. These findings expanded the previous observation of different genetic background and offered potential target signaling cascades for treating EBER‐positive DLBCL.

**FIGURE 2:**
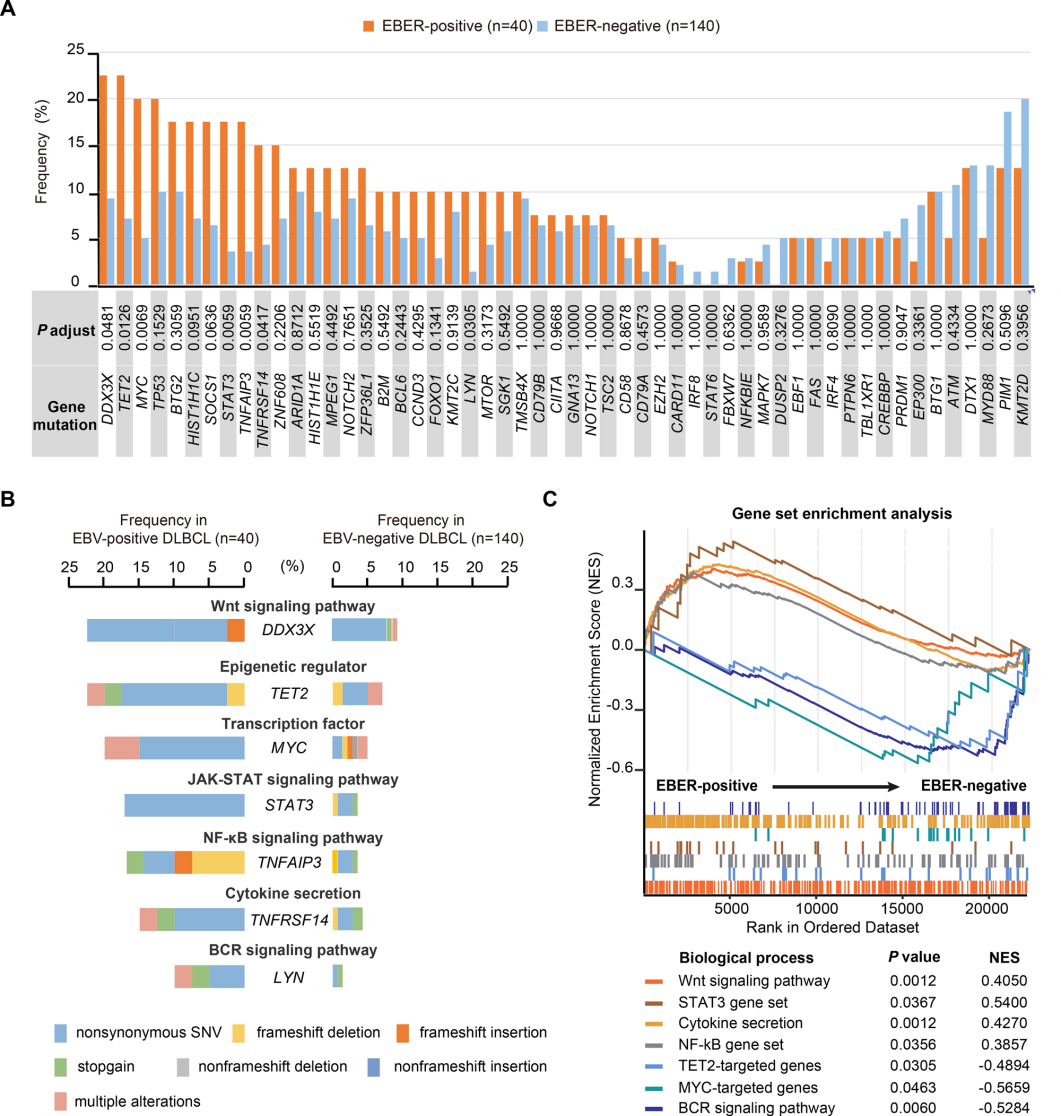
Mutation analysis in DLBCL according to tumor EBER. (A) Bar graph showed the prevalence of gene mutations in 54 lymphoma‐associated genes, along with the *P* value for the difference in prevalence between 40 EBER‐positive patients and 140 EBER‐negative patients. (B) Bar graph showed the prevalence and type of recurrent mutations in EBER‐positive patients. Mutation types are color‐coded as indicated in the legend below. (C) Gene‐Set Enrichment Analysis (GSEA) revealed pathway alterations in 12 EBER‐positive patients, as compared to those in 32 EBER‐negative patients. GSEA preranked tool was used to analyze STAT3 gene set and NF‐κB gene set, as well as *MYC*‐targeted and *TET2*‐targeted genes (Table S7)

As revealed by CNV analysis in 122 patients with WGS/WES data, EBER‐positive patients represented a distinctive pattern of copy number alterations, involving frequently deletions (>10%) in chromosome 6p21.32‐33, 8p23.1, and 6p22.1, as well as recurrent amplifications in chromosome 1q32.1 and 1q21.3 (Figure 3A). Interestingly, genes in deletion regions (> 10%) were mostly involved in processing and presentation of endogenous peptide antigen (*P* < 0.0001, Figure 3B). Given the impact of loss of antigen processing and presentation on antitumor immunity,^8^ we observed a significant relationship between antigen processing and presentation *Z*‐score and Immune Score (*P* = 0.0003, *n* = 200; Figure 3C), obtained from RNA‐sequencing data by ssGSEA and xCell method. Moreover, EBER‐positive DLBCL presented significantly decreased antigen processing and presentation *Z*‐score and Immune Score, as compared to EBER‐negative DLBCL. Specifically, we focused on the interactions between MHC class I/II molecular genes and immune cell subtypes, ranked by their association strengths with Immune Score or antigen presentation and processing Z‐score (Figure 3D). MHC class I‐related molecules (*B2M*, *HLA‐C*, *HLA‐B*, *NLRC5*, *MR1*, and *CD74*) were significantly decreased in EBER‐positive patients and correlated with dendritic cells (DC) and gamma delta/CD4+/CD8+ T‐cell subtypes. Corresponding decreases in CD4+memory T‐cells (*P* = 0.0084), total CD8+ T‐cells (*P* = 0.0002), CD8+effector memory T‐cells (CD8+Tcm, *P* = 0.0005) and CD8+ central memory T‐cells (CD8+Tem, *P* = 0.0499), were also observed in EBER‐positive DLBCL (Figure 3E). As MHC class I molecules play a critical role in presenting viral or tumor antigen for CD8+ cytotoxic T‐cell activation,^9^ loss of MHC class I molecules could allow for persistent EBV infection and impair tumor immunosurveillance in EBER‐positive DLBCL. Thus, it might be implications for immunotherapies targeting antigens, such as EBV‐specific T cell therapy, in treating EBER‐positive DLBCL.^10^

**FIGURE 3:**
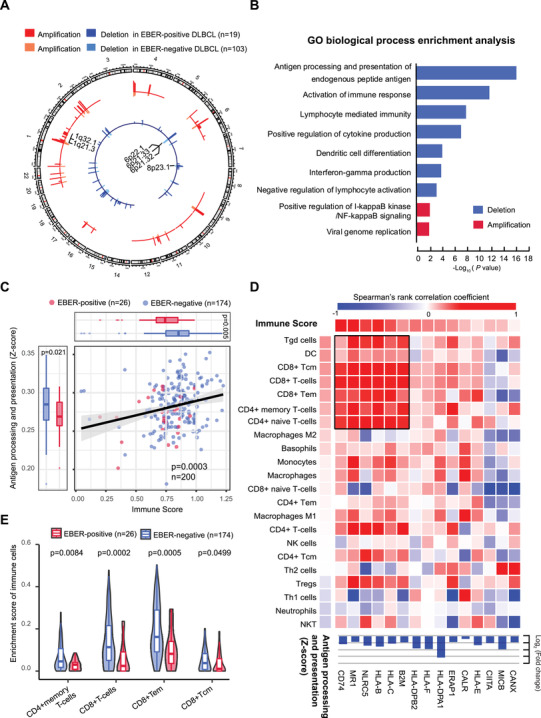
Copy number variation analysis in DLBCL according to tumor EBER. (A) Circos plot showed the frequencies of recurrent copy‐number alterations in 19 EBER‐positive patients (outside of circle), as compared with 103 EBER‐negative patients (inside of circle). (B) GO biological process enrichment analysis of genes in deletion (blue) or amplified (red) genomic regions in EBER‐positive DLBCL. (C) Correlation between antigen presentation and processing Z‐score and Immune Score using Spearman's rank correlation. Upper and left panels indicate two scores stratified by 26 EBER‐positive patients (red) and 173 EBER‐negative patients (blue). (D) Heatmap shows Spearman's correlation coefficients of MHC class I/II molecular genes expression with immune cell enrichment scores. Bars on the lower side of the heatmap indicate fold changes in the expression of MHC I/II molecular genes significantly downregulated in EBER‐positive DLBCL. (E) Enrichment scores of indicated immune cells in 26 EBER‐positive patients (red) and 173 EBER‐negative patients (blue). *P* values comparing difference in two groups are indicated above the columns. DC, dendritic cell; Tcm, central memory T cell; Tem, effective memory T cell; Tgd cell, gamma delta T cell; NK, Natural killer cell; Treg, T regulatory cell

In conclusions, EBV contributed to tumor progression in DLBCL with distinct oncogenic mutations and tumor microenvironment alterations.

## CONFLICT OF INTEREST

All authors declare that no conflict of interest exists.

## AUTHORS' CONTRIBUTIONS

C.‐X.Z. and J.‐J.W. collected and analyzed clinical data, prepared biological samples, analyzed the omics data and wrote the article. D.F. and P.‐P.X performed the experiments and analyzed the omics data. S.C., L.W., X.W., J.‐F.Z., L.‐P.S., Z.‐W.C., J.‐P.C., M.‐Y.F., T.L., Y.‐P.S., K.Y., Y.L., J.G. and M.H. gathered detailed clinical information for the study and analyzed the data. C.‐F.W. conducted pathological analysis. W.‐L.Z., and J.‐D.H. conceived the study, directed, and supervised research and wrote the manuscript.

## Supporting information

SUPPORTING INFORMATION

SUPPORTING INFORMATION

SUPPORTING INFORMATION

SUPPORTING INFORMATION

SUPPORTING INFORMATION

SUPPORTING INFORMATION

SUPPORTING INFORMATION

SUPPORTING INFORMATION

SUPPORTING INFORMATION
