# Supplementary material for: Clinical and molecular features of Epstein‐Barr virus‐positive diffuse large B‐cell lymphoma: Results in a multi‐center trial
Source: Clin Transl Med. 2021 Sep 16;11(9):e539. doi: 10.1002/ctm2.539 (PMC8444560; doi:10.1002/ctm2.539)
Supplement: Supplementary file 1 — SUPPORTING INFORMATION [file CTM2-11-e539-s009.pdf]

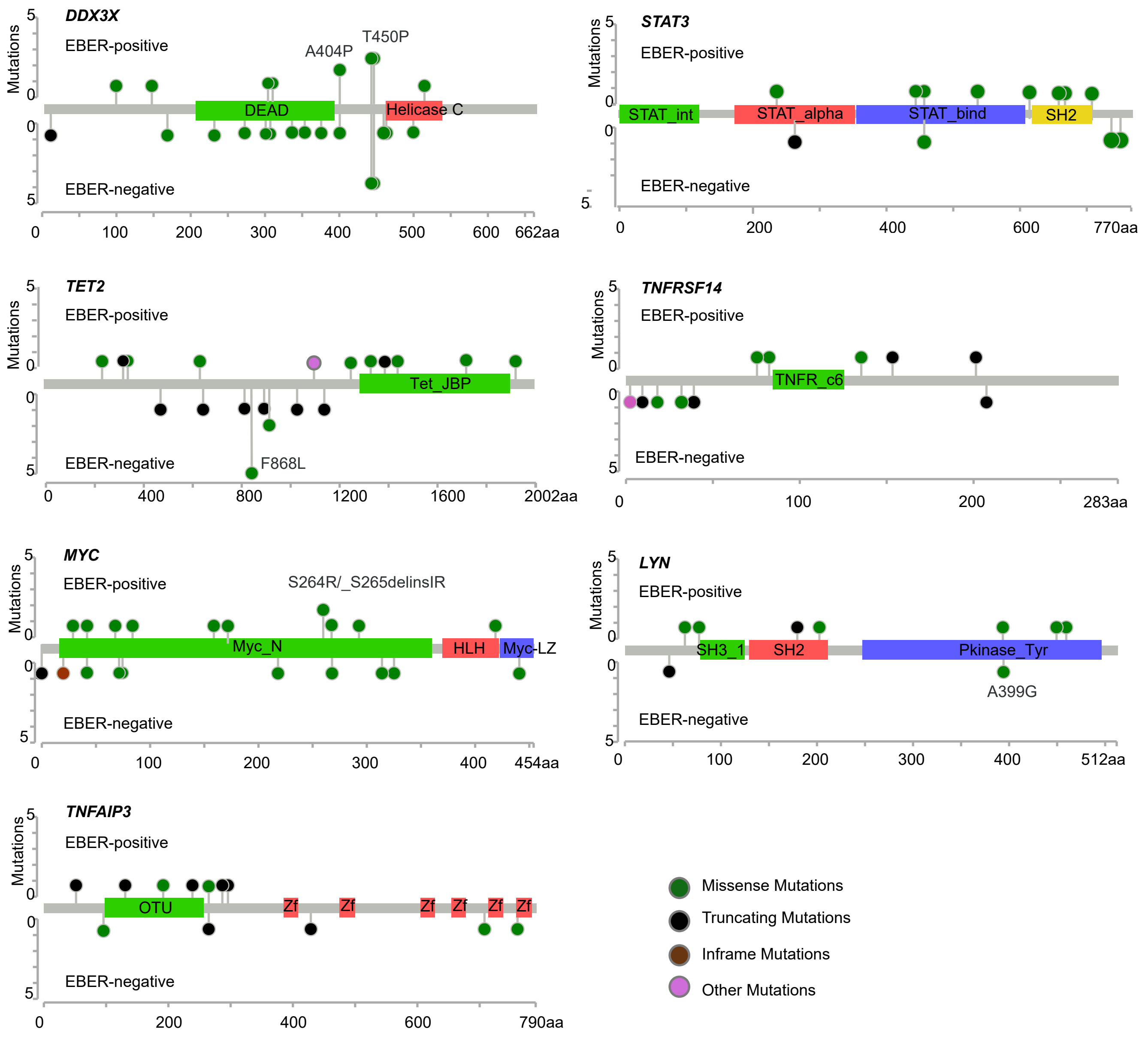

Figure S1. Position of mutational alterations in the gene structures of *DDX3X*, *TET2*, *MYC*, *TNFAIP3*, *STAT3*, *TNFRSF14* and *LYN*. Diagram showing mutations identified in the protein locus Domains of protein identified in this report are indicated by horizontal lines. Mutation diagram circles are colored with respect to the corresponding mutation types. In case of different mutation types at a single position, color of the circle is determined with respect to the most frequent mutation type. Tet_JBP, Oxygenase domain of the 2OGFeDO superfamily; DEAD, DEAD/DEAH box helicase; Helicase_C, Helicase conserved C - terminal domain; Myc_N, Myc amino-terminal region ; HLH, Helix-loop-helix DNA -binding domain; Myc-LZ, Myc leucine zipper domain; OUT, OTU-like cysteine protease; zf-A20, A20-like zinc finger; STAT_bind, STAT protein, DNA binding domain; STAT_int, STAT protein, protein interaction domain; STAT_alpha, STAT protein, all alpha domain ; TNFR_c6, TNFR/NGFR cysteine -rich region ; Pkinase_Tyr, Protein tyrosinekinase.
