# Supplementary material for: Clinical and molecular features of Epstein‐Barr virus‐positive diffuse large B‐cell lymphoma: Results in a multi‐center trial
Source: Clin Transl Med. 2021 Sep 16;11(9):e539. doi: 10.1002/ctm2.539 (PMC8444560; doi:10.1002/ctm2.539)
Supplement: Supplementary file 9 — SUPPORTING INFORMATION [file CTM2-11-e539-s003.doc]

**Supplemental Methods**

**Patients**

Between May 15, 2013 and March 16, 2016, patients with newly diagnosed DLBCL, as defined by 2008 WHO criteria^1^, were enrolled from 20 centers of the Multicenter Hematology/Oncology Programs Evaluation System (M-HOPES) in China. Patients ≤60 years were randomly treated with six cycles of R-CHOP50, R-CEOP70 or R-CEOP90 (rituximab 375mg/m2 intravenously on day 0, cyclophosphamide 750mg/m2, doxorubicin 50mg/m2 [R-CHOP50] or epirubicin 70mg/m2 [R-CEOP70] or epirubicin 90mg/m2 [R-CEOP90], and vincristine 1.4mg/m2 (maximum dose, 2mg) intravenously on day 1, and prednisone 60mg/m2 [maximum dose, 100mg] orally from day 1 to day 5). Patients >60 years were randomly assigned to receive six cycles of R-CHOP50 or R-CEOP70. All patients were treated with two additional courses of rituximab at 375mg/m^2^. The study was approved by the Review Board of all M-HOPES Hospitals with informed consent obtained in accordance with the Declaration of Helsinki. Among all, 429 patients had available tumor samples and 141 patients had available serum samples. Table S5 describe the patient’s selection for the clinical or genomic analysis.

**Immunohistochemistry and in-situ hybridization**

Immunohistochemistry was performed on 5 μm-paraffin sections with an indirect immunoperoxidase method. Germinal center B-cell (GCB) or non-GCB subgroups were determined using Hans classification^3^, with 30% cut-off value of CD10, BCL-6, and MUM-1. EBER in-situ hybridization was performed on local medical centers, according to central assessment defined by Pathologist Chaofu Wang and Xiao chun Fei at 20% cut-off value ^4^ of EBER-positive cells on 3-μm-paraffin sections.

**EBV DNA load quantification and EBV serology**

EBV DNA was extracted from serum using the QIAamp DNA Mini Kit (Qiagen, Valencia, CA, USA) and quantified by EBV PCR Fluorescence Quantitative Diagnostic Kit (Da An Gene Co, Sun Yat-sen University, China) with a cut-off value of 100 copies/ml. Levels of EBV specific antibodies were measured in serum, using quantitative ELISA Kit (DiaSorin, Saluggia, Italy) of EBNA-1 IgG and EA IgG, qualitative ELISA Kit of VCA IgA (Euroimmun, Lubeck, Germany) and Rta IgG (Tarcine BioMed Inc, Beijing), with threshold serum activity of 20.0 IU/ml.

**Sample preparation**

Genomic DNA was extracted from frozen tumor tissue using a QIAamp DNA Mini Kit (Qiagen, Hilden, Germany) and from formalin-fixed paraffin-embedded (FFPE) tumor tissue samples using a GeneRead DNA FFPE Tissue Kit (Qiagen, Hilden, Germany). Total RNA was extracted from frozen tumor tissue using Trizol and RNeasy Mini Kit (Qiagen, Hilden, Germany). Genomic analysis was conducted on 180 DLBCL patients with available sequencing data, including 161 patients in the NHL-001 trial and 19 EBER-positive patients form the Shanghai Ruijin Hospital. There was no significant difference of baseline characteristics and gene mutation signatures in the EBER-positive patients in the NHL-001 trial or in Ruijin cohort (Table S6). Sequencing data have been deposited at the National Omics Data Encyclopedia (NODE, https://www.biosino.org/node/) under accession number OEP0001143. Reviewer link can be sent to specified e-mail address if required.

**Identification of somatic mutation**

**Sequencing and alignment.** For whole genome sequencing (WGS), Covaris DNA shearing system was used for shearing genomic DNA to about 300bp fragments. After end-repair and 3’-ends adenylation, DNA fragments were ligated with Illumina PE adapters to generate the indexed library. Agilent 2100 Bioanalyzer was applied to validate the library and sequencing was carried out on Illumina HiSeq platform with 150bp paired-end strategy in WuXi Next CODE, Shanghai. For whole extron sequencing (WES), SeqCap EZ Human Exome kit (version 3.0) was used to capture exome regions and sequencing was performed on HiSeq 4000 platform with 150bp paired-end strategy in YuanQi, Shanghai. By Burrows-Wheeler Aligner (BWA) version 0.7.13-r1126, read pairs were aligned to RefSeq hg19 (downloaded from UCSC Genome Browser, URLs). Samtools version 1.3 was applied to generate chromosomal coordinate-sorted bam files and to remove PCR duplications. Then the reads were realigned around potential indel regions using Genome Analysis Toolkit (GATK) version 3.4 IndelRealigner with the recommended pipeline. Of each sample, the mean depth was 120.23× (range 50~200×), with an average 98.34% (range 81.70%~99.83%) of the target sequence which had been covered sufficiently deep for variant calling (≥10× coverage).

**SNV/indel calling and filter workflow.** SNVs and indels were called by GATK Haplotype Caller and GATK Unified Genotyper. The homemade pipeline was applied for filtering SNVs and indels which had been detected by the above software. Primary exclusion criteria for the filtering were 1) mutations reported with low confidence; 2) germline mutations detected from control samples; 3) mutations reported in 1000 Genomes (dbSNP 137) as common SNPs and not included in COSMIC version v77.

**SNV/indel annotation.** Using the UCSC Genome Browser (http://genome.ucsc.edu/) and Refseq database (Human Reference Genome version hg19), SNVs and indels were mapped to the genome location. All the somatic functional mutations were obtained, including 1) nonsynonymous SNVs; 2) frameshift or in-frame indels; 3) stop-gain or stop-loss. Potential false positive results were eliminated by visual inspection.

**Targeted sequencing.** Based on the WES and WGS results, we identified 135 recurrent and functional mutated genes constituting the targeted sequencing panel with two criteria: (i) genes and gene families with highly recurrent mutations (>5%) in DLBCL and (ii) genes associated with oncogenesis according to the existing literature. Fifty-eight patients with FFPE tumor tissue quality-controlled by agarose electrophoresis were included for extended validation by targeted sequencing. Primer 5.0 software was used to design PCR primers. Multiplexed libraries of tagged amplicons from tumor tissue samples were generated by Shanghai Yuanqi Bio-Pharmaceutical Multiplex-PCR Amplification System. Using established Illumina protocols, we performed deep sequencing on HiSeq 4000 platform (Illumina).

**Identification of mutation spectrum in Chinese DLBCLs**

Recurrent (at least 3 times) and functional gene mutations were identified in 179 patients with both WES/WGS and transcriptome sequencing data based on three criteria: (i) genes related to oncogenesis of DLBCL according to the existing literature, (ii) genes involved in key biological processes and (iii) functional mutations with significant gene expression alternations or clinical significance. In total, recurrent and functional mutations in 54 genes were observed in 175 of the 179 patients. Genomic data have been deposited at the National Omics Data Encyclopedia (NODE, http://www.biosino.org/node) under accession number OEP0001143. Reviewer link can be sent to specified e-mail address if required.

**Copy number analysis**

CNVkit was applied to copy number analysis of WGS and WES samples, using the batch pipeline recommended in the CNVkit manual (https://cnvkit.readthedocs.io/en/stable/pipeline.html). Sequencing reads were mapped to human reference sequence GRCh37 by BWA (version 0.7.17) and deduplicated by Picard (version 2.23.3). GATK (version 4.0.0) was then used to correct sequencing depth profile in terms of GC-content, capture target size and regions containing sequence repeats. The copy number profile of each tumor sample was quantified by log2 ratio of reads compared to the normal samples. As for significant gained or lost copy number aberrations (CNAs), CNAs for genes were identified by Genomic Identification of Significant Targets in Cancer (GISTIC, version 2.0) with default parameters and calculated for focal homozygous deletions or high-level amplifications referring to a log2 ratio thresholds of ±0.8.

**Oncogenic pathway analysis**

RNA-seq was performed using Illumina HiSeq 2000 (Illumina, California, USA) at the depth of 20-40 million 150 bp paired-end reads per sample. The HTSeq (http://www.htseq.org/) was applied to generate transcript counts table files. Package ‘limma’ (v3.38.3)^5^was performed to normalize the raw reads and obtain differentially expressed genes (DEGs). Normalized reads were analyzed by package ‘clusterProfiler’ (v3.10.1)^6^ to run Gene-set enrichment analysis (GSEA)^7^ and Gene Ontology enrichment analysis with DEGs. GSEA preranked tool was used to analyze STAT3 and NF-κB gene set, as well as *MYC*-targeted and *TET2*-targeted genes (Table S7)

**Estimation of gene signatures and immune cell subtype.**

To estimate the performances of antigen presentation and processing, single-sample gene set enrichment analysis (ssGSEA) algorithm (R package ‘gsva’)^8^ were used to quantify the Z-score of Gene Ontology biological process (antigen presentation and processing)^9^ for single sample available for RNA sequencing data in Rong Shen et al. cohort^10^.

To further determine the profile of tumor immune microenvironment, xCell^11^, a computational method, was applied to estimate the scores of immune cell type enrichment and overall score of immune cells (Immune Score). Briefly, the ssGSEA scores are calculated for 489 gene signatures. According to the characteristics of cell development and differentiation, scores of a cell type are averaged for corresponding gene signatures. Finally, the enrichment score of 34 types of immune cells, was obtained for each sample.

Molecular classification DLBCL genotypes were identified using the LymphGen probabilistic classification tool (R code version, <https://doi.org/10.5281/zenodo.3700087>). Genetic aberrations including mutations, copy number alterations, and fusions were analyzed and integrated. The probabilities of each GenClass-defined genotype were calculated in 122 patients with WES/WGS data. Identified patients were assigned into six genotypes (MCD, BN2, N1, EZB, ST2, and A53) according to the classification tool.

**Statistical analysis**

Progression-free survival (PFS) was measured from entry onto a study until lymphoma progression or last follow-up. Overall survival (OS) time was calculated from the date of diagnosis to death or last follow-up. Survival curves were plotted by the Kaplan-Meier method. The Cox proportional hazards regression model was constructed for univariate and multivariate analysis. P values were calculated by appropriate statistical tests (Fisher’s exact test or Log-rank test) using SPSS software, version 22.0. P value of <0.05 (two-sided) was considered statistically significant. Correlations between normalized expression of MHC I/II molecular genes and immune cell subtype infltration were assessed by Spearman’s correlation analysis. In multiple comparisons for gene mutations, P values were adjusted by Bonferroni correction.

**References**

1. SH S e. WHO classificaiton of tumors of haematopoietic and lymphoid tissues.: World Health Organization classification of tumors,4th edn;. 2008;

2. Cunningham D, Hawkes EA, Jack A, et al. Rituximab plus cyclophosphamide, doxorubicin, vincristine, and prednisolone in patients with newly diagnosed diffuse large B-cell non-Hodgkin lymphoma: a phase 3 comparison of dose intensification with 14-day versus 21-day cycles. *Lancet*. May 25 2013;381(9880):1817-26. doi:10.1016/S0140-6736(13)60313-X

3. Hans CP, Weisenburger DD, Greiner TC, et al. Confirmation of the molecular classification of diffuse large B-cell lymphoma by immunohistochemistry using a tissue microarray. *Blood*. 2015;103(1):275-282.

4. Park S, Lee J, Ko YH, et al. The impact of Epstein-Barr virus status on clinical outcome in diffuse large B-cell lymphoma. *Blood*. Aug 01 2007;110(3):972-8. doi:10.1182/blood-2007-01-067769

5. Ritchie ME, Phipson B, Wu D, et al. limma powers differential expression analyses for RNA-sequencing and microarray studies. *Nucleic acids research*. Apr 20 2015;43(7):e47. doi:10.1093/nar/gkv007

6. Yu G, Wang LG, Han Y, He QY. clusterProfiler: an R package for comparing biological themes among gene clusters. *Omics : a journal of integrative biology*. May 2012;16(5):284-7. doi:10.1089/omi.2011.0118

7. Subramanian A, Tamayo P, Mootha VK, et al. Gene set enrichment analysis: A knowledge-based approach for interpreting genome-wide expression profiles. *Proceedings of the National Academy of Sciences*. 2005;102(43):15545-15550. doi:10.1073/pnas.0506580102

8. Hänzelmann S, Castelo R, Guinney J. GSVA: gene set variation analysis for microarray and RNA-seq data. *BMC bioinformatics*. Jan 16 2013;14:7. doi:10.1186/1471-2105-14-7

9. Liberzon A, Birger C, Thorvaldsdóttir H, Ghandi M, Mesirov JP, Tamayo P. The Molecular Signatures Database (MSigDB) hallmark gene set collection. *Cell systems*. Dec 23 2015;1(6):417-425. doi:10.1016/j.cels.2015.12.004

10. Shen R, Xu PP, Wang N, et al. Influence of oncogenic mutations and tumor microenvironment alterations on extranodal invasion in diffuse large B-cell lymphoma. *Clinical and translational medicine*. Nov 2020;10(7):e221. doi:10.1002/ctm2.221

11. Aran D, Hu Z, Butte AJ. xCell: digitally portraying the tissue cellular heterogeneity landscape. *Genome biology*. Nov 15 2017;18(1):220. doi:10.1186/s13059-017-1349-1
